# An internet survey of 2,596 people with fibromyalgia

**DOI:** 10.1186/1471-2474-8-27

**Published:** 2007-03-09

**Authors:** Robert M Bennett, Jessie Jones, Dennis C Turk, I Jon Russell, Lynne Matallana

**Affiliations:** 1 Oregon Health & Science University, Portland, OR, USA; 2 California State University, Fullerton, USA; 3 University of Washington, Seattle, WA, USA; 4 The University of Texas Health Science Center at San Antonio, TX, USA; 5 National Fibromyalgia Association, Orange, CA, USA

## Abstract

**Background:**

This study explored the feasibility of using an Internet survey of people with fibromyalgia (FM), with a view to providing information on demographics, sources of information, symptoms, functionality, perceived aggravating factors, perceived triggering events, health care utilization, management strategies, and medication use.

**Methods:**

A survey questionnaire was developed by the National Fibromyalgia Association (NFA) in conjunction with a task force of "experts in the field". The questionnaire underwent several rounds of testing to improve its face validity, content validity, clarity and readability before it was mounted on the internet. The questionnaire consisted of 121 items and is available online at the website of the National Fibromyalgia Association.

**Results:**

The questionnaire was completed by 2,569 people. Most were from the United States, with at least one respondent from each of the 50 states. Respondents were predominantly middle-aged Caucasian females, most of whom had FM symptoms for ≥ 4 years. The most common problems were morning stiffness, fatigue, nonrestorative sleep, pain, concentration, and memory. Aggravating factors included: emotional distress, weather changes, insomnia, and strenuous activity. Respondents rated the most effective management modalities as rest, heat, pain medications, antidepressants, and hypnotics. The most commonly used medications were: acetaminophen, ibuprofen, naproxen, cyclobenzaprine, amitriptyline, and aspirin. The medications perceived to be the most effective were: hydrocodone preparations, aprazolam, oxycodone preparations, zolpidem, cyclobenzaprine, and clonazepam.

**Conclusion:**

This survey provides a snap-shot of FM at the end of 2005, as reported by a self-selected population of people. This descriptive data has a heuristic function, in that it identifies several issues for further research, such as the prescribing habits of FM health care providers, the role of emotional precipitants, the impact of obesity, the significance of low back pain and the nature of FM related stiffness.

## Background

Over the past 20 years fibromyalgia (FM) has emerged as a leading cause of office visits to rheumatologists, both in its primary form and as an accompaniment of other rheumatic disorders. Epidemiological studies report a FM prevalence of between 2 and 7% in most nations [1-12], with a female to male ratio of approximately 9:1. It is increasingly evident that FM represents a significant challenge in view of its high prevalence, frequent comorbidities, and frustration with current treatment modalities.

Patient self-report is increasingly used to assess the impact of rheumatic diseases and gain insight into their impact and to formulate new questions for investigation [13-16]. Study groups such as OMERACT (Outcome Measures in Rheumatology) use self-reported information as a preliminary step in the development of Delphi-based questionnaires with the eventual aim of defining optimal outcome measures for clinical trials [17].

The major aims of the current study were to conduct a large internet-based national survey of people with FM to determine: (1) demographics and sources of information about FM, (2) symptoms and functionality, (3) perceived aggravating factors, (4) perceived triggering events, (5) diagnosis and health care utilization, (6) management strategies, and (7) medication use.

## Methods

### Questionnaire development

This questionnaire was developed in conjunction with The National Fibromyalgia Association (NFA) and a multidisciplinary Task Force of FM experts. The NFA is a nonprofit organization whose mission is to develop and execute programs dedicated to improving the quality of life for people with FM by increasing the awareness of the public, media, government, and medical community. The first draft of the questionnaire was completed in 2004 with a focus group of 12 FM volunteers providing feedback.

In order to establish content validity of the Questionnaire the following steps were taken:

• The questionnaire was administered to 42 people (ages 24–74) diagnosed with FM who attended a symposium on FM that was held at California State University, Fullerton. Based on the resulting feedback, the questionnaire was modified to improve its content, clarity, readability, and overall quality.

• The revised questionnaire was further tested on the a focus group of 21 people with FM (age range 36–60) who had volunteered to participate in NFA surveys via e-mail. Based on feedback from this focus group, the Questionnaire was further adapted to further improve its content, clarity, readability, and quality.

• A 2-day workshop of the Task Force met in October 2005 to finalize the Questionnaire content and format.

• A study was conducted to determine whether the finalized Questionnaire had appropriate stability over a 1–2 week period. Intraclass correlation coefficients (ICCs) were calculated to index test-retest reliability. The majority of the single ICC values fell within the acceptable range of values from 0.82 to 1.00 with few exceptions. Most items with lower ICCs required participants to recall specific dates or to estimate things which occurred within a specified time frame (i.e., recall issues). Others items with lower ICCs related to symptoms and states (i.e., clinical features that typically fluctuate in intensity and frequency).

The reading level of the Questionnaire, except for section # 6 (medications), was estimated by using the "readability statistics" tool that is available in Microsoft Word. This analysis showed a Flesch Reading Ease score of 60.8 and a Flesch-Kincaid Grade Level of 7.0. The grade level estimates range from grade 5 through college; grade 7 is fairly low; indicating the Questionnaire was quite suitable for use by lay people.

The final Questionnaire consisted of 121 items [18] and consisted of 5 sections:

(1) Background Information (questions 1–25), (2) Symptoms (questions 26–51), (3) Physical Ability Impact (questions 52–63), (4) Employment Impact (questions 64–87), and (5) nature and effectiveness of interventions (questions 88–121). A decision was made to post the survey on the Internet for whatever period was required to obtain a representative sample of approximately 3,000 responses.

## Results

The questionnaire was posted on the NFA's website on October 12, 2005. The survey was completed in a satisfactory manner by 2,569 people over the course of 3 days. The vast majority of responses were from the United States. There was at least 1 responder from each of the 50 States in the United States. In most States approximately 0.05% of the estimated female FM population responded to the survey -based on a conservative estimate FM prevalence of 3.5% in women and the 2002 National Census figures [19]. In addition, there were 46 responses from 12 other countries (Canada, United Kingdom, Ireland, Australia, New Zealand, Mexico, Germany, Holland, Bermuda, France, Israel, and Hungary).

### Demographics and the sources of information about FM

The demographics of the population responding to the survey are shown in Table 1. The responders were predominantly middle-aged Caucasian females, 75% of whom had experienced FM symptoms for more than 4 years. Only 3.2% of the responders were male. The age distribution was slightly skewed towards older individuals (mean = 47.3 ± 10.68, range 17–77.5) which is consistent with published research on FM. They tended to be moderately overweight and reported having gained approximately 50 pounds since they were aged 18. Seventy percent had a BMI > 25 and 43% had a BMI > 30; comparable BMI figures for white females taken from National Health Interview Survey of 2004 are 47% and 21% respectively [19]. Just over 50% of the responders had a household income of between $20,000 and $80,000.

Respondents obtained information about FM from diverse sources, including professional and consumer organizations: Health care professionals providing FM information were: family physicians (45.8%), rheumatologist (43.6%), internists (23.1%), massage therapists (20.3%), chiropractors (20.2%), physical therapists (14.4%), mental health professionals (psychiatrist/psychologist/social workers) (13.1%), pharmacists (7.8%), nurse practitioners/physician assistants (7.6%), nurses (5.3%), nutritionist/dietitians (4.4%), and gynecologists (2.9%). In addition to health care professionals, respondents received information from a number of other sources including: NFA (sponsors of the survey,70%), general media (41.6%), Arthritis Foundation (35.2%), Internet message boards (23.4%), Internet chat rooms (12.5%), local support groups (12%), and informal sources (e.g., friends,32.6%), health food store,13.6%, family member,10.7%). These percentages do not summate to 100% as more than one source could be acknowledged.

### Symptoms and functionality

FM patients are usually poly-symptomatic with symptoms and syndromes affecting several organ systems. Every respondent in this survey reported multiple current symptoms and syndromes that have been associated with FM. The most common symptom was low back pain followed by recurrent headaches, arthritis, muscle-spasm, tingling, and balance problems (Table 2). The average intensity of symptoms over the past week was captured in response to the question "please select the number that best describes your experience with the following (*specified problem*) on average during the past week". This questions was followed by asking the respondents to evaluate each symptom on a scale of 0 (no symptom) to 10 (extreme symptom). The highest rated symptoms were: morning stiffness, fatigue, poor sleep, and pain. A considerable number of cognitive symptoms (i.e., problems with memory and concentration) were also acknowledged (Table 3).

Chronic pain, fatigue stiffness, and other FM-associated symptoms frequently impact on an individual's functional capabilities. Survey participants responded to the question: "even if you did not do the following activities, please indicate what you think your ability to do them during the past week would be?" The percentage of responders who indicated that they would have *no difficulty *performing each task was as follows: normal activities of daily living (65%); walking 2 blocks (45%); climbing stairs (38%); shopping (34%); light household duties (e.g., cooking/dusting) (32%); lifting or carrying 10 pounds (30%); walking half a mile (27%); walking 1 mile (18%); lifting or carrying 25 pounds (8%); heavy household duties (e.g., vacuuming/scrubbing) (7%); and strenuous recreational activity (hiking/biking) (3%).

The respondents were nearly equally divided regarding their ability to maintain gainful employment. Those who were still working felt that their symptoms compromised their ability to be productive due to frequent absences and reduced work hours. Approximately 20% of the respondents had filed some form of disability claim and 6% received workman's compensation.

### Perceived aggravating factors

People with FM typically report that various events exacerbate their symptoms. These attributions were probed with the question "which of the following factors act as triggers that worsen your symptoms?" The respondents were permitted to list as many as they wished and thus the percentages do not sum to 100. The most common exacerbating events acknowledged were mental stressors, weather changes, sleeping problems and strenuous activity (Table 4).

### Perceived triggering events

People with FM often associate a specific event to the onset of their symptoms. In response to the question "have any of the following potential triggering events occurred around the same time that your fibromyalgia symptoms first became apparent" Approximately 21% of responders indicated that they could not identify any such association. Over 73% of those who indicated some triggering event made attributions to emotional trauma or chronic stress (Table 5). The next most common attribution was acute illness (26.7%), followed by physical stressors (surgery, motor vehicle collisions, and other injuries). Another 20.6% of the responses acknowledged physical or emotional abuse as a child and 15.1% associated abuse as an adult to the onset of their symptoms. Childhood sexual abuse was cited by 9% of respondents. Approximately 10.1% of the responders related the onset of FM symptoms to the menopause.

### Diagnosis and health care utilization

Most responders (98.4%) had been formally diagnosed as having FM by a health care provider. In 55% of responders the diagnosis had been made 4 or more years prior to the completion of the survey, whereas in 9% the diagnosis had been made within the last year. Rheumatologists were responsible for the diagnosis in 42.4% of responders followed by family physicians and internists in 23.2% and 12.2%, respectively. In just under 10% of responders the diagnosis of FM was made by other health care providers (chiropractors, nurse-practitioners, osteopaths, psychiatrists, gynecologists, and nurses). Some 46% had consulted between 3 to 6 health care providers before obtaining the diagnosis of FM, while 24.6% had seen >6 health care providers prior to diagnosis. In response to the question "how legitimate does your current health care provider treat your fibromyalgia?", there was roughly a bimodal distribution with 65.1% reporting "legitimate" or "very legitimate", whereas 27.8% felt that their health care provider did *not *view FM as a "very legitimate" disorder.

Over 96% of responders had seen a health care provider at least once over the past year for their FM symptoms. Some 44% of responders reported that they had visited a health care provider 1 to 4 times in the past year. An additional 23% indicated they visited responder 5 to 8 times in this time period, 14% reported 9 to 12 visits, and 13% reported more than 12 visits (13%). Approximately 29% of respondents indicated they had visited an emergency room on at least 1 to 4 occasions in the last year. Hospitalization due to FM symptoms was reported by 3.5% of the responders.

Approximately 70.4% of the sample reported that they had medical insurance. Most responders had out-of-pocket expenses related to management of their FM. For instance 74% of responders reported spending between $100 and $500 each month on over-the-counter products; whereas 61% spent between $100 and $500 each month on prescription medications.

### Management strategies

Survey participants were asked to "indicate whether you use any of the following interventions for FM and if so, whether each helps your FM symptoms". Respondents rated the effectiveness of each intervention on a 0 to 10 scale, with 10 being most effective The interventions perceived to be most effective (effectiveness rating ≥ 6.0) in descending order were: rest, heat modalities, prescription pain medications, prescription antidepressants, prescription sleep medications, prayer, massage, and pool therapy (Table 6).

### Medications used by survey responders

The Questionnaire listed 253 medications and asked "which of the following medications do you currently use, or have tried in the past to relieve symptoms due to fibromyalgia and were they helpful?" Respondents rated the effectiveness of each intervention as "being helpful" (= 10) or "not helpful" (= 0). Table 7 presents the results in 4 columns: "every used ", " use now ", "continuing use " (computed from (use now/every used) ×100), and "considered helpful ". The most commonly used medications (*ever used*) were acetaminophen, nonsteroidals (NSAIDs), tricyclic antidepressants, and cyclobenzaprine. The most helpful medications (*considered helpful*) were: hydrocodone preparations, aprazolam, oxycodone preparations, zolpidem, clonazepam, cyclobenzaprine, and codeine preparations. The medications used most consistently used over time (*continuing use*) were: hydrocodone preparations, ibuprofen, clonazepam, acetaminophen, trazodone, gabapentin, and zolpidem.

## Discussion

Over the course of 3 days, over 2500 respondents completed a survey that was posted on the web site of the National Fibromyalgia Association. The demographics of the respondents to this survey revealed no particular surprises when compared to other epidemiologic studies and surveys [1-12]. The household income was similar to a previous survey of 537 FM patients in the San Diego, California [20]. In comparison to United States National Census figures, respondents were moderately overweight. Several other studies have reported obesity in FM [21-23] and weight reduction has been reported to provide improvement [24]. This concordance in demographic variables with other published FM studies suggests that the Questionnaire provided a reasonably representative measure.

It is difficult to make direct comparisons of health care utilization in this survey and other studies, as it is largely derived from patients with internet access in the United States. For example, a Canadian study reported annual visits to health care providers were 11.6, emergency room visits were 0.6, and hospital inpatient days or 2.1 [25]. However, the health care system in Canada is a nationalized service compared to the fee for service system in the United States. It is impossible to disentangle the availability and costs of services from service utilization.

Although a hallmark of FM is pain, the respondents listed morning stiffness, fatigue, and non-restorative sleep before pain, as comparable in severity. While morning stiffness is a common symptom in inflammatory rheumatic disorders [26,27] it has only been mentioned in the occasional FM study [28,29]. Morning stiffness is a characteristic feature of inflammatory arthritidies, where it has been defined as "slowness or difficulty moving the joints when getting out of bed or after staying in one position too long, which involves both sides of the body and gets better with movement" [30]. Whether such a precise definition would apply to FM patients would be an interesting topic for future study. Morning stiffness in rheumatoid arthritis has been correlated with elevated serum levels of hyaluronic acid [31]. Raised levels of hyaluronic acid have been reported in one study FM patients [32], but not in several others [33-35]. The 63% prevalence of low back pain is particularly noteworthy, as this has not been previously reported in survey or epidemiological studies of FM. It may be a productive topic for future research, as patients with low back pain have been reported to develop FM in 25% of cases [36] and some patients with LBP have augmented pain processing [37].

Six of the most frequently cited exacerbating factors involved some form of emotional distress (endorsed by 83% of the respondents). These results are in accord with several other studies [38-40]. The perceived adverse effect of weather changes (80% endorsed) is noteworthy. This effect has been previously reported [41] but in 2 studies FM symptoms failed to correlate with meteorological variables [42,43]. In another study pain was correlated with high atmospheric pressure and low temperature [44]. Concordant with several other reports [45-47] "forgetfulness" and problems with "concentration" were rated as quite common and problematical. The frequency of disability claims in the current survey was about the same as a multicenter study in the United States [48].

One study of the prevalence of sexual abuse in FM suggested that it might be as high as 57% [49]. The 7.8% prevalence of childhood sexual abuse in the current survey is substantially lower and may reflect differences in "candidness" between an Internet based survey population and one-on-one interviews. A recent telephone based study found no association between FM and sexual abuse, but did report a three-fold increase of FM in women who had been raped. Thus, it was hypothesized that chronic stress, in the form of posttraumatic stress disorder, may mediate the relationship between rape and FM [50].

Among the most interesting data to come out of this survey was the use of various management strategies and medications by the participants. An attempt was also made to assess the perceived effectiveness of various therapies (on a 0 to 10 scale, with 10 being most effective). The 3 most commonly used interventions were non-medicinal (rest, heat, and distraction). Interventions with the highest effectiveness ratings were: prescription sleep medications (rating: 6.5), prescription pain medications (rating: 6.3), resting (rating: 6.3), heat (rating: 6.3), and prescription antidepressants (rating: 6.2). Although non-prescription pain medications and nutritional supplements were commonly used they did not appear to be particularly effective (ratings of 3.8 and 2.8 respectively). Conversely, pool therapy, used by a quarter of the respondents, was as effective as many of the more frequently used management strategies.

The most commonly used medications were acetaminophen (94%), ibuprofen (87%), naproxen (66%), cyclobenzaprine (64%), and amitriptyline (55%). Based on the percentage of respondents who rated medications as helpful, the top 10 were: hydrocodone preparations (75%), aprazolam (70%), oxycodone preparations (67%), diazepam (65%), zolpidem (64%), clonazepam (61%), cyclobenzaprine (58%), codeine preparations (55%), propoxyphene preparations (54%), and ibuprofen (51%). Interestingly, there is a discrepancy between the most commonly used and the most effective medications. This, discrepancy may be associated with the heavy use of over-the-counter drugs, which are generally cheaper than prescription drugs. There may also be a reluctance of physicians to provide ongoing prescriptions of opioids and benzodiazepines. The perceived effectiveness of hydrocodone preparations is of some interest as this medication has never been formally tested in FM patients. Future research should examine the profile of health care providers who prescribed this and similar opioids. Aprazolam (reported as being helpful in 70% of respondents) has been noted to be of some benefit when used in conjunction with ibuprofen [51], but has never been formally evaluated as a stand-alone drug in FM.

Another way to consider effectiveness of medications is to examine continuation of treatment. The most commonly reported medications that respondents *continuing *to use were: hydrocodone preparations (41%), ibuprofen (41%), clonazepam (40%), acetaminophen (37%), gabapentin (36%), trazodone (36%), zolpidem (34%), aprozolam (30%), cyclobenzaprine (30%), and bupropion (29%). As expected, newer medications available at the end of 2005, such as duloxetine, sodium oxybate, and pregabalin, were being used by only a small percentage of respondents (i.e., < 8%). Non-adherence to prescribed medications is reported to be common in FM patients [52], but whether this is a result of lack of efficacy, side effects, cost, or psychosocial factors, is not known.

In interpreting the results of this survey it is important to acknowledge its limitations. The surveyed population was self-selected as people with FM who had Internet access and was familiar with the NFA website. Approximately 70% of the respondents indicated that they obtained information about FM from the NFA (the sponsors of the survey). It is possible that those familiar with NFA differ in important ways from people with FM in general. They were not personally interviewed or formally diagnosed. Thus an unknown proportion of those responding may not have met in 1990 ACR classification criteria for a diagnosis of FM [53]. However, only 1.6% of the responders reported that their diagnosis of FM had not been confirmed by a health care professional. There is some evidence that certain combinations of symptoms are strongly correlated with an ACR based diagnosis of FM. For instance, Katz has reported that a history of widespread regional pain (score ≥ 8.0) plus fatigue (score ≥ 6.0) has a 75% concordance with ACR criteria [54]. This Internet based survey was new and previously untested and there is a need to confirm its psychometric properties in future studies. Preliminary testing suggested good face validity and test-retest reliability. Internet-based surveys are increasingly used to obtain patient data [55] and several studies have reported a good correlation with paper-and- pencil surveys [56,57].

## Conclusion

The information from this survey provides as a glimpse into the FM landscape in the United States at the end of 2005 as perceived by a self-selected group of FM patients with Internet access. These results pose several issues for more in depth research, such as the prescribing habits of FM health care providers, the role of emotional precipitants, the impact of obesity, the significance of low back pain and the nature of FM related stiffness.

## Competing interests

The National Fibromyalgia Association received an unrestricted educational grant from Pfizer, Inc. for support of this study. Pfizer Inc. had no role in implementing the design of this study; in the collection, analysis, and interpretation of data; in the writing of the manuscript; or in the decision to submit the manuscript for publication.

## Authors' contributions

RMB provided the statistical analysis and was responsible for writing the manuscript with help and advice from the co-authors.

JJ did the initial work in developing the questionnaire and provided help with the methods

DCT participated in the statistical analysis and provided help with the results and discussion

IJR participated in result analysis and the discussion

LM initiated this project, provided the website used in the survey and provided editing advice

## Pre-publication history

The pre-publication history for this paper can be accessed here:



## Figures and Tables

**Table 1:** Demographic profile of responders

**Demographic Feature**	**Frequency**
Age [years]	47.3 ± 10.68
Female	96.8%
Current weight [pounds]	179.5 ± 45.9
Weight at age 18	129.1.5 ± 26.8
Height [inches]	64.7 ± 3.2
Body mass index [BMI]	30.1 ± 7.6
Symptoms < 7 months	0.6 %
7–12 months	1.5%
1–2 years	3.9%
2–4 years	13.1%
> 4 years	75.5%
Race/Ethnicity: Caucasian	91.5%
Afro-American	2.3%
American Indian	3.6%
Hispanic	2.6%
Asian	0.4%
Marital Status: Currently married	64.2%
Never married	11.2%
Divorced	17.4%
Widowed	2.5%
No children	26.5%
Number of Children: One child	16.5%
Two	30.5%
> 3 children	20.9%
Household income: < $9,000	4.9%
$10,000–$19,999	8.7%
$20,000–$29,999	8.8%
$30,000–$39,999	10.6%
$40,000–$49,999	10.4%
$50,000–$59,999	9.8%
$60,000–$69,999	7.5%
$70,000–$79,999	6.2%
$80,000–$89,999	5.2%
$90,000–$99,999	5.1%
$100,000–$199,999	9.7%
> $200,000	1.7%

**Table 2:** Frequencies of symptoms and current comorbidities ≥ 25 % *[in descending order of frequency]*

**Current Symptom**	**Frequency**
Low back pain	63%
Recurrent headaches	47%
Arthritis	46%
Muscle spasm	46%
Tingling	46%
Balance problems	45%
Irritable bowel syndrome	44%
Numbness	44%
Chronic fatigue	40%
Bloating	40%
Depression	40%
Anxiety	38%
Sinus problems	37%
Tooth disorders	32%
Restless legs	32%
Tinnitus	30%
Jaw pain	29%
Bladder problems	26%
Rashes	25%

**Table 3:** Symptom intensity during the past week *[in descending order of severity]*

**Symptom**	**Mean ± SD**
Morning stiffness	7.2 ± 2.5
Fatigue	7.1 ± 2.1
Nonrestorative sleep	6.8 ± 2.7
Pain	6.4 ± 2.0
Forgetfulness	5.9 ± 2.7
Concentration	5.9 ± 2.6
Difficulty falling asleep	5.6 ± 3.3
Muscle spasms	4.8 ± 3.2
Anxiety	4.5 ± 3.1
Depression	4.4 ± 3.1
Headaches	4.3 ± 3.1
Anger	3.9 ± 2.9
Restless legs	3.6 ± 2.7
Abdominal pain	3.6 ± 2.8
Poor balance	3.5 ± 2.9
Swelling of feet and ankles	3.2 ± 3.1
Dizziness	2.9 ± 2.8
Bladder problems	2.5 ± 2.9
Skin rashes	1.9 ± 2.9

**Table 4:** Factors perceived to worsen FM symptoms *[in descending order of frequency]*

**Perceived stressor**	**Frequency**
Emotional distress	83%
Weather changes	80%
Sleeping problems	79%
Strenuous activity	70%
Mental stress	68%
Worrying	60%
Car travel	57%
Family conflicts	52%
Physical injuries	50%
Physical inactivity	50%
Infections	43%
Allergies	37%
Low to moderate physical activity	36%
Lack of emotional support	36%
Time zone changes	34%
Airplane travel	34%
Perfectionism	32%
Work related conflicts	29%
Menses	27%
Medication side effects	27%
Chemical exposures	27%
Sexual intercourse	17%

**Table 5:** Perceived triggering events of FM onset *(in descending order of frequency)*

**Event**	**Frequency**
Chronic stress	41.9%
Emotional trauma	31.3%
Acute illness	26.7%
Physical injury (non-MVA)	17.1%
Surgery	16.1%
Motor vehicle accident (MVA)	16.1%
Emotional and physical abuse as an adult	12.2%
Emotional and physical abuse as a child	11.9%
Thyroid problems	10.3%
Menopause	10.1%
Sexual abuse as a child	8.7%
Childbirth	7.6%
Sexual abuse as adult	2.9%

**Table 6:** Interventions used by the survey responders *[in descending order of frequency]*

**Intervention**	**Frequency**	**Effectiveness [0–10 scale]**
Resting	86%	6.3 ± 2.5
Distraction [reading, watching TV etc.]	80%	4.7 ± 2.5
Heat modalities [warm water, hot packs]	74%	6.3 ± 2.3
Nutritional supplements	68%	3.8 ± 2.8
OTC pain medications	67%	3.8 ± 2.3
Prescription pain medications	66%	6.3 ± 2.4
Gentle walking	64%	4.6 ± 2.6
Prescription antidepressants	63%	6.2 ± 2.8
Stretching	62%	5.4 ± 2.6
Prayer	57%	6.0 ± 2.9
Prescription sleep medications	52%	6.5 ± 2.7
Relaxation/meditation	47%	5.1 ± 5.5
Massage/reflexology	43%	6.1 ± 2.8
Aerobic exercise	32%	5.0 ± 3.0
Cold therapy [ice packs etc.]	30%	4.8 ± 2.8
Chiropractic manipulation	30%	5.1 ± 3.0
Counseling [psychologist, MSW, pastor]	29%	4.8 ± 3.0
Pool therapy	26%	6.0 ± 3.0
Non-aerobic exercise [stretching, yoga, Tai Chi]	24%	5.1 ± 2.9
Physical therapy	24%	4.7 ± 3.1
OTC sleep medications	22%	4.0 ± 2.9
TENS unit	21%	4.3 ± 2.9
Trigger point injections	21%	5.0 ± 3.3
Support groups	19%	4.6 ± 3.0
Strength training	18%	4.3 ± 2.9
Pain clinic	17%	4.8 ± 3.1
Acupuncture	15%	4.5 ± 3.5
Pilates	8%	4.6 ± 3.3
Cognitive behavioral therapy	8%	4.3 ± 3.2
Energy healing [e.g. Reiki]	7%	4.0 ± 3.2
Biofeedback	6%	2.9 ± 2.9
Spinal surgery	4%	3.4 ± 3.4
Hypnosis	3%	2.5 ± 2.9

**Table 7:** Medications used by ≥ 25% of the survey responders [% use sorted on "ever used"]

**Drug (with common USA brand name)**	**Ever used**	**Use now**	**Continuing use**	**Considered helpful**
Acetaminophen [Tylenol]	94	35	37	36
Ibuprofen [Motrin, Advil]	87	36	41	51
Naproxen [Naprosyn, Aleve]	66	13	20	39
Cyclobenzaprine [Flexeril]	64	19	30	58
Amitriptyline [Elavil]	55	12	22	42
Aspirin [Ecotrin]	53	10	19	34
Celecoxib [Celebrex]	48	6	13	40
Rofecoxib [Vioxx]*	48	0	0	39
Codeine + APAP [Tylenol #2 or #3 or #4]	47	4	9	55
Tramadol [Ultram]	46	13	28	44
Hydrocodone + APAP [Vicodin]	44	18	41	75
Propoxyphene + APAP [Darvocet]	44	8	18	54
Zolpidem [Ambien]	41	14	34	64
Sertraline [Zoloft]	41	8	20	40
Fluoxetine [Prozac]	39	8	21	42
Paroxetine [Paxil]	36	4	11	32
Buproprion [Wellbutrin]	35	10	29	41
Trazadone [Desyrel]	33	12	36	51
Gabapentin [Neurontin]	33	12	36	46
Aprazolam [Xanax]	33	10	30	70
Oxycodone + APAP [Percocet, Roxicet]	32	7	22	67
Venlafaxine [Effexor]	30	8	27	44
Melatonin [Melatonex]	28	5	18	35
Diazepam [Valium]	28	3	11	65
Tramadol + APAP [Ultracet]	27	7	26	49
Clonazepam [Klonopin]	25	10	40	61
Valdecoxib [Bextra]*	25	0	0	40

* No longer available in the USA
